# Innovative Materials for Construction

**DOI:** 10.3390/ma13235448

**Published:** 2020-12-02

**Authors:** Mariaenrica Frigione, José Luís Barroso de Aguiar

**Affiliations:** 1 Innovation Engineering Department, University of Salento, Prov.le Lecce-Monteroni, 73100 Lecce, Italy; 2 Civil Engineering Department, University of Minho, Campus de Azurém, 4800-058 Guimarães, Portugal; aguiar@civil.uminho.pt

Academic and industrial efforts around the world are continuously engaged to develop new smart materials that can provide efficient alternatives to conventional construction materials and improve the energy-efficiency in buildings or are able to upgrade, repair, and protect existing infrastructures. This new generation of materials, before an actual market entry, needs to be analyzed, validated, tested on in the field, and, possibly, modeled to enable predictions as to their long-term behavior and performance. To this regard, the valuable contributions in the Special Issue “Innovative Materials for Construction” provide a collection of original research and new trends in the field of innovative materials and technologies proposed for the construction sector, with a special focus on sustainable materials for the building industry of the future, keeping attention on innovative solutions suitable for ancient constructions and cultural heritage.

The first stage of any construction design relies on the selection of the best-performing materials able to satisfy the project goal. Although this is a very important task in the design of a construction project, since mechanical, functional, and physical properties of construction materials greatly affect the overall project performance, there are currently no systematic methods to guide designers in this selection. The work by Lee and co-workers [1] would propose an original approach to fill this gap based on an analytic hierarchy process (AHP) method. The authors validated the AHP method in the design of a composite system form (CSF) panel, proving the efficacy of the proposed model able to identify the best combination of materials and ensuring the greatest performance.

In concrete technology, the shrinkage occurring during the cure of concrete represents a major problem, hampering appropriate performance and limiting durability of concrete structures. To address this issue, Masanaga et al. [2] investigated the introduction of a new shrinkage-reducing agent (SRA) to a concrete mix based on Portland cement, comparing its properties and performance with those of a conventional SRA. The superiority of the new shrinkage-reducing agent was confirmed in terms of improved durability of concrete towards freeze-thaw cycles. Additionally, the authors of the study analyzed the mechanism, through which the proposed SRA was able to reduce shrinkage in concrete.

The incorporation of industrial by-products in construction materials is a sustainable and convenient way to exploit waste to produce new materials, eliminating the problem of waste treatment and, at the same time, avoiding the use and depletion of new natural resources. To this regard, the research presented by Terrones-Saeta et al. [3] proposed the incorporation of ladle furnace slag in reclaimed asphalt pavements (RAPs) in the cold in-place recycling, with the bitumen emulsion manufacturing technique. The authors demonstrated that, with a proper selection of the asphalt mix components (RAP, ladle furnace slag, water, and emulsion), it is possible to manufacture road pavements characterized by good mechanical properties and durability.

The development of interior mortars, based on different binders (aerial and hydraulic lime, gypsum and cement), containing an original sustainable phase change material (PCM) able to improve the energy efficiency of buildings, was the object of two publications resulting from an Italian–Portuguese scientific collaboration [4,5]. Since PCMs have the ability to change their physical state according to the environmental temperature, the incorporation of a suitable PCM in mortars can reduce indoor temperature fluctuations, leading to improvement in human comfort and reductions in energetic consumption. The selection of the components to produce a “green” composite PCM system was based on the use of non-toxic, low environmental impact materials, and waste materials/by-products from other industries. The authors of this study demonstrated that it was possible to obtain mortars with suitable mechanical properties with an optimization of their composition, depending on the kind of binder [4]. Subsequently, the measurements of thermal performance of the best-performing mortars, i.e., those based on cement and gypsum, confirmed that the addition of the experimented PCM in mortars leads to a decrease of the maximum achievable temperatures in the hot season and an increase of the minimum temperatures in the cold season, with a reduction of heating and cooling indoor needs, thus confirming the capability of this new PCM material to achieve energy savings.

The microbial-induced calcium carbonate precipitation (MICP) has received great attention for its potential in construction applications. This technique has been used in biocementation of sand, consolidation of soil, production of self-healing concrete or mortar, and removal of heavy metal ions from water. The self-healing ability is very important for repair of concrete structures. The presence of bacteria is essential for MICP to occur. To this regard, the paper presented by Chuo et al. [6] reviews the bacteria used for MICP in some of the most recent studies. Several factors that affect MICP performance are bacterial strain, bacterial concentration, nutrient concentration, calcium source concentration, addition of other substances, and methods to distribute bacteria. The paper presented by Imran et al. [7] proposed the addition of plant-based natural jute fibers to MICP-treated sand. The results of this study showed that the added jute fibers improved the engineering properties (ductility, toughness, and brittleness behavior) of the biocemented sand using the MICP method. The fibers facilitated the MICP process by bridging the pores in the calcareous sand, reduced the brittleness of the treated samples, and increased the mechanical properties of the biocemented sand. The results of this study could significantly contribute to further improvement of fiber-reinforced biocemented sand in geotechnical engineering field applications.

The study of materials that can influence the cement hydration is very significant. These admixtures modify the hydration behavior, contributing to the adaptation of the cementitious materials to the site conditions. Madej [8] showed that the substitution of Sr^2+^ for Ca^2+^ in the Ca_7_ZrAl_6_O_18_ lattice decreased the reactivity of Sr-substituted Ca_7_ZrAl_6_O_18_ in the presence of water. Therefore, strontium can be considered an inhibition agent for cement hydration. The techniques NMR, XRD, and SEM-EDS were employed for the justification of this behavior. The authors studied calcium zirconium aluminate cement pastes and detected important changes during the first 24 h or 7 days of hydration.

The use of fiber reinforced polymer (FRP) technologies is gaining great success worldwide as an efficient alternative to steel reinforcements to improve load-bearing and performance of modern constructions, as well as ancient buildings. The complete study of these materials should include experimental and numerical analysis.

Chin and co-workers [9] developed rebars with headed ends with the aim to improve their anchorage to concrete. The authors of this study systematically evaluated the mechanical properties of FRP rebars and the bond strength developed with concrete, and these parameters were fundamental in the design of FRP structural members. Pull-out tests were performed by changing the GFRP rebar diameter, the concrete strength, and the head type; precast concrete decks connected with different headed GFRP rebars were also tested in flexural mode to estimate the flexural behavior of the connected decks. The results confirmed the effectiveness of the headed GFRP rebars, evidencing the effects of the different parameters analyzed.

Kopecki et al. [10] used a non-linear numerical analysis of the examined structures by comparing them with the results of the model experiment. The study contains the results of the experimental research using models made of glass epoxy composites. The results of the research allowed the creation of the concept of an adequate numerical model in terms of the finite element method, allowing to determine the distribution of stress and strain in the components of the studied structures. The presented research allows to determine the nature of the deformation of composite thin-walled structures, in which local loss of stability of the covering is acceptable in the area of post-critical loads.

The mechanical behavior (in flexural, tensile, and compressive modes and the energy fracture) of lime-based mortars reinforced by randomly oriented short glass fibers, with different contents and aspect ratios, was investigated by Angiolilli et al. [11]. The aim of their research was the assessment of the effect of the introduction of various types/amounts of fibers in binders, reproducing the compositions of historical lime-based mortars. The obtained results highlighted that the fiber reinforced mortar composites ensures increased strength and excellent ductility capacity, thus making them a promising alternative to traditional fiber reinforcement systems, even for ancient constructions.
